# Evaluation of surgical margin status in patients with early glottic cancer (Tis-T2) treated with transoral CO_2_ laser microsurgery, on local control

**DOI:** 10.1007/s00405-018-5070-9

**Published:** 2018-07-19

**Authors:** Martine Hendriksma, Marc W. Montagne, Ton P. M. Langeveld, Maud Veselic, Peter Paul G. van Benthem, Elisabeth V. Sjögren

**Affiliations:** 1 Department of Otorhinolaryngology, Head and Neck Surgery, Leiden University Medical Center, Leiden, The Netherlands; 2 Department of Pathology, Leiden University Medical Center, Leiden, The Netherlands

**Keywords:** Local control, Surgical margins, Laser surgery, Early glottic cancer, Wound bed biopsy

## Abstract

**Purpose:**

To assess the impact of surgical margins status on local control in patients with primary early glottic (Tis-T2) squamous cell carcinoma after treatment with transoral CO_2_ laser microsurgery (TLM) and to assess the significance of additional wound bed biopsies.

**Methods:**

Patients with Tis-T2 tumours treated with TLM type I–III resections according to the European Laryngological Society classification between 2009 and 2013 were included in retrospective analysis. Recurrence rate was determined in patients with free versus non-free specimen margins and wound biopsies. Five-year survival rates were determined using the Kaplan–Meier method. Prognostic impact of pT-category, resection margin status, tumour differentiation, wound bed biopsy status, and number of biopsies on local control (LC) were tested with the log-rank test.

**Results:**

Eighty-four patients were included in the analysis. Positive margins were seen in 68 patients (81.0%). Margin status after TLM did not significantly influence LC (*p* = 0.489), however, additional wound bed biopsies were significantly associated with lower LC (*p* = 0.009). Five-year LC, disease-specific survival, overall survival and laryngeal preservation were 78.6, 78.0, 98.6 and 100%, respectively.

**Conclusions:**

Additional wound bed biopsies can help predict local recurrence in patients treated with TLM for early glottic carcinoma. We propose that there is enough evidence to support a wait-and-see policy in patients with positive specimen margins and negative wound bed biopsies. For patients with positive wound bed biopsies, further treatment is warranted.

## Introduction

Early glottic cancer (Tis-T2) is a highly treatable disease with high local control rates (LC), for treatment with either transoral CO_2_ laser microsurgery (TLM) or radiotherapy (RT). Several studies did not identify significant differences in local control between TLM and RT for Tis-T2 tumours [1–6].

To assess elimination of cancerous cells after TLM, pathologic evaluation of the surgical specimen is common practice. The aim in early glottic carcinoma is to perform a narrow margin resection in order to preserve as much tissue as possible to maximize preservation of laryngeal functions [7–9]. The surgical margins ideally should be free of neoplastic cells, with a layer of healthy cells surrounding the excised tumour.

It is commonly acknowledged that the assessment and interpretation of surgical margins in TLM have some issues. First of all the use of laser leads to the evaporation of tissue and charring of the specimen thus demolishing the actual surgical margin by approximately 0.05–0.5 mm [8, 10, 11]. Therefore, it is not uncommon to find close or positive margins during final pathology examination [7, 12]. Currently, literature still shows no clear definition of negative, close or positive surgical margins [13] and recommendations for free margins vary from 0.5 to 2 mm [7, 8, 10, 11, 14–17]. Moreover, controversy remains over the interpretation of surgical margins because of difficulties with orientation after piecemealing, the small size of the specimens, tissue retraction as a result of thermal energy on elastic fibres, thermal damage, and charring. Furthermore, fixation of tissue induces shrinkage, which also has to be taken into account on pathologic evaluation.

In literature, some studies conclude that positive margins have a negative impact on oncological outcome [14, 18–20] and patients should thus be retreated. However, others conclude that positive margins have no influence on local control [8, 10, 11, 16]. Therefore, the best management of close or positive margins has not been clearly determined [13] although it has been shown that mandatory retreatment could lead to unnecessary additional treatment in up to 84% of these patients [11]. Therefore, a wait-and-see policy could also be considered appropriate restricting the number of needless procedures performed [10]. According to the latest ELS recommendation for follow-up of laryngeal cancer, a second-look microsurgery is however mandatory in cases of positive surgical margins [21]. Nonetheless, some authors suggest that a second-look is not required in all patients and propose that with experience and good clinical judgement a philosophy of watch and wait is also appropriate [22]. Therefore, the role of second-look microsurgery is still debatable.

Other techniques that can be used to improve surgical margins assessment are frozen section analysis, optical and molecular imaging techniques of which narrow-band imaging (NBI) is the most widely implemented, and wound bed biopsies. Frozen section analysis is a reliable, cost-effective method preventing routine second-look procedures [7, 23], although it does require extra operating time for every patient. Furthermore, intraoperative NBI can help in better defining surgical margins and reduce positive surgical margins, although this technique is only helpful for the mucosal plane [24, 25]. For the past years, in an attempt to obtain more certainty about the radicality of our resections while not extending operating time, we have used additional wound bed biopsies in our institution to guide further management. Therefore, the objective of this study was to assess the impact of surgical margins status on local control in patients with primary early glottic (Tis-T2) squamous cell carcinoma (SCC) after treatment with TLM and to assess the significance of wound bed biopsies.

## Materials and methods

Records of all patients treated for early glottic cancer (Tis-T2) at the Leiden University Medical Centre (LUMC) between January 2009 and December 2013 were retrieved. Early glottic cancer was defined as tumour stage Tis, T1a, T1b or T2 with a fully mobile vocal fold without lymph node involvement or metastasis (N0M0) at the start of treatment. The medical charts of these patients were retrospectively assessed and patients receiving TLM with a curative intent were included. Patients with previous laryngeal cancer or other treatments were excluded. Data on patient demographics, pathologic T-category, follow-up, additional treatments and patient outcomes were collected. Pathology reports were assessed for: status of the resection margin, tumour differentiation, additional wound bed biopsy status, and number of biopsies.

Before TLM, all but 15 patients were staged by endoscopy and had biopsy-proven SCC. Two patients had frozen section analysis during their first endoscopy and thirteen patients were clinically suspicious. All these patients had TLM in the same session. Transoral CO_2_ laser microsurgery was carried out under general anaesthesia using a Sharplan laser with a digital acublade micromanipulator typically set in continuous or continuous superpulse mode. In most cases, the tumour was first transected to assess the depth of tumour invasion and then resected in two pieces. Resections varied from type I to type III of the European Laryngological Society (ELS) classification, as during the study period tumours requiring larger resections were treated by RT according to Dutch guidelines. Tumour specimens were pinned on a piece of cork and were sent to the pathologist for histological examination accompanied by descriptive drawings. In most cases standard practice was followed and additional wound bed biopsies were taken and contained separately. Typically, the wound bed biopsies were taken at five different points: four at the edges of the tumour ground and one deep biopsy in the middle of the tumour ground. If surgeons found it necessary, extra biopsies could be taken.

For the sake of this study pathological specimens and pathology reports were reviewed by the pathologist (MV). We defined surgical margin status as follows: not free if SCC or severe dysplasia was found in the margins, free if no SCC or severe dysplasia was found in the margins or not assessable if the assessment was impossible due to artefacts made either during or after surgery. We defined the wound bed biopsy status as follows: a positive biopsy was defined as one or more biopsies containing SCC or severe dysplasia; negative biopsies contained no SCC or severe dysplasia tissue, but could contain mild or moderate dysplasia; not taken meant that in that operation, no wound bed biopsies were taken.

Generally, the patients were discharged on the same day of surgery. All patients were followed according to protocol with flexible fiberoptic laryngoscopy scheduled every 2 months in the first year and with decreasing frequency afterwards until 5 years. In case of positive margins or wound biopsies, a second-look procedure or re-resection could be planned or regular follow-up was scheduled depending largely on the surgeon’s clinical evaluation of the procedure.

### Statistical methods

Statistical analysis was performed with SPSS version 23.0 (Armonk, NY: IBM Corp.). Categorical variables were described using frequency and percentages. Means and standard deviations were reported for descriptive statistics. The entry point was the date of TLM. The endpoint for local control (LC) was the date of the first local recurrence. The endpoint for disease-specific survival (DSS) was the date of death, due to laryngeal cancer. Patients who died of unrelated or unknown causes were considered as without recurrence at the date of death. The endpoint for overall survival (OS) was to the date of death (all causes) or last follow-up. The endpoint for laryngeal preservation (LP) was the date of laryngectomy. Observations were censored at 60 months. Median follow-up was calculated with the reverse Kaplan–Meier method and given with the 95% CI. Survival analysis was performed with the Kaplan–Meier method to calculate LC, DSS, OS, and LP. The prognostic value of five categorical variables (pT-category, resection margin, tumour differentiation, additional wound bed biopsy results, and biopsy count) on local control was tested by univariate analysis with the log-rank test. For the pT-category, T1a and T1b are grouped, because the group with T1b tumours was too small. A *p* value of < 0.05 was considered statically significant.

## Results

### Patients

**Fig. 1:**
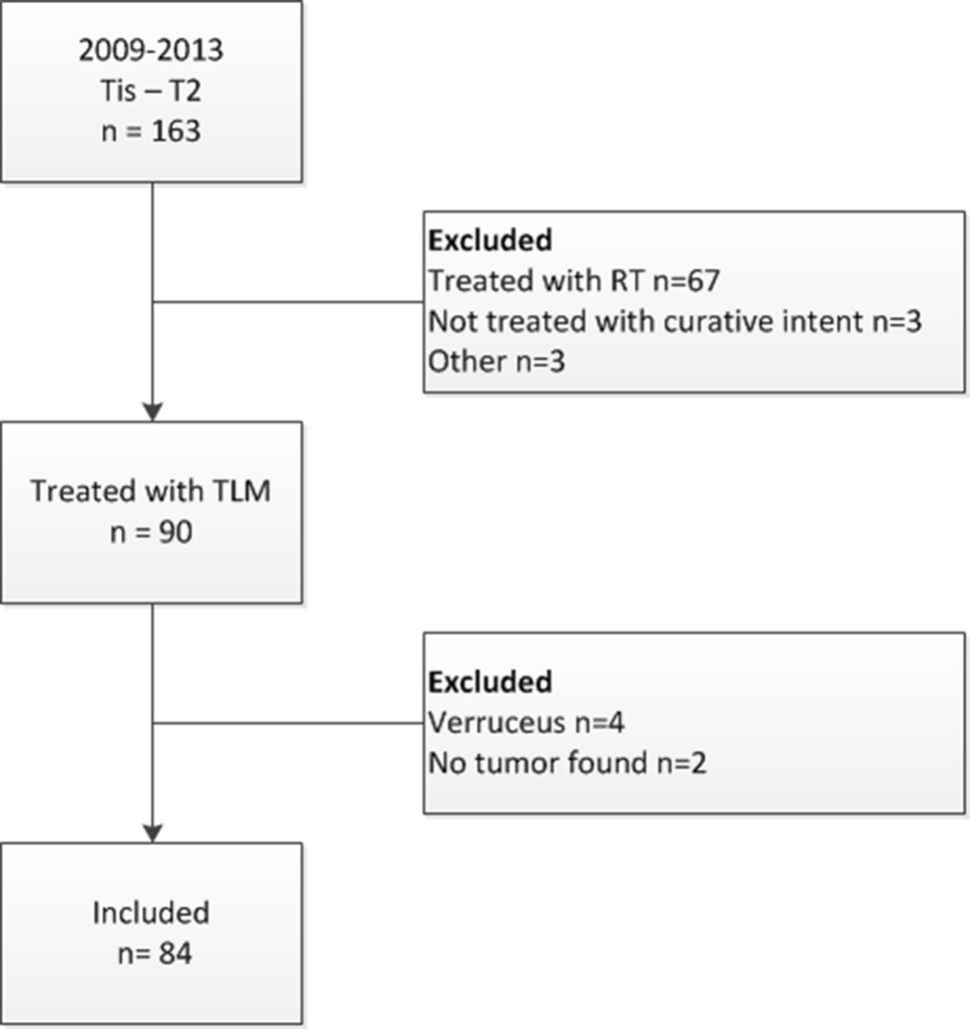
Flow diagram

Between 2009 and 2013, 163 consecutive patients suffering from early glottic carcinoma (Tis-T2N0M0) with mobile vocal folds, were treated with either TLM or RT at our centre. Of these, 90 cases were primarily treated with TLM with curative intent. The remaining 73 patients were excluded; 67 of them were primarily treated with radiotherapy, 3 received TLM but without curative intent (debulking as part of overall curative treatment), 1 patient had microlaryngeal surgery without laser, and 1 patient had a laryngectomy performed because of a history of another primary glottic carcinoma treated with RT. Finally, one patient underwent laser surgery in spite of the protocol suggesting laryngectomy, in an attempt to preserve the larynx. This patient had a history of RT for oropharyngeal cancer, and the lesion normally would have been considered too advanced for TLM. Eventually, cancer recurred with laryngectomy as a result after all. Out of the 90 patients treated with curative intent with TLM, 4 cases were excluded on the basis of histology (verrucous carcinoma) and in 2 cases no malignancy was found. Eighty-four cases were therefore included in this study (Fig. 1). One patient developed a second primary early glottic carcinoma (stage T1a on the contralateral vocal fold) in the timespan of this study and was therefore included twice. Patients’ demographics are listed in Table 1. Median follow-up was 53.0 months (95% CI 50.3–55.7 months). Three patients were lost to follow-up after 40.3 months and one patient moved after 51 months and had regular follow-up elsewhere. All patients had assessable surgical margins.

**Table 1:** Baseline characteristics

Characteristics	No. of cases (%) Total = 84 (100%)
Mean age at surgery (SD)	68.7 (9.3)
Sex	
Male	75 (89.3)
Female	9 (10.7)
Pathologic T category	
Tis	19 (22.6)
T1a	45 (53.5)
T1b	5 (6.0)
T2	15 (17.9)
Surgical margin status	
Not free	68 (81.0)
Free	16 (19.0)
Wound bed biopsies	
Positive	12 (14.3)
Negative	55 (65.4)
Not taken	17 (20.2)

### Surgical margins

The surgical margins were negative in 16 patients (19.0%). None of these patients underwent further treatment. Two patients with negative surgical margins (12.5%) developed local recurrence. Surgical margins were positive in 68 patients (81.0%). In this group, 12 patients (17.6%) had second-look microsurgery with re-excision. Only 3 patients (25.0%) showed evidence of persistent carcinoma after this re-excision. None of the patients were treated with RT because of positive margins. Overall, 14 patients with positive margins (20.6%) developed a local recurrence (Table 1).

### Wound bed biopsies

In 67 patients additional wound bed biopsies were taken, with a mean of 4.3 (SD 3.2) biopsies per person. In 17 patients (20.2%) no additional wound bed biopsies were taken. The wound bed biopsies were negative in 55 patients (82.1%) (Table 1). Seven patients (12.7%) with negative wound biopsies developed a recurrence. The wound bed biopsies were positive in 12 patients (17.9%). All these patients had both mucosal and deep biopsies, of which in 11 patients superficial biopsies were positive and in 1 patient a deep biopsy was positive. Four of these patients underwent re-excision without showing evidence of residual disease. None of the patients with positive wound bed biopsies were treated with RT. Overall, 5 patients (41.7%) with positive wound bed biopsies developed local recurrence of whom 2 had undergone a re-resection. In the patients without additional wound bed biopsies, 4 patients developed recurrent disease (23.5%). In patients of this group with re-resections, 2 patients (50%) developed recurrent disease nonetheless.

### Survival

During follow-up, 16 patients developed a local recurrence (19.0%), after a median of 5.0 months (95% CI 2.4–7.6 months). Five patients (31.3%) were treated with TLM again, 8 patients (50.0%) received RT, 2 patients (12.5%) received TLM plus RT and 1 patient (6.3%) required a total laryngectomy, but refused this treatment and was therefore treated with supportive care. Three patients developed a second local recurrence after a median of 9 months (95% CI 0.0–20.2 months). Two of them were once more treated with TLM and the other patient received RT. No patients developed metastasis or regional lymph nodes. During follow-up, 22 patients (26.2%) died, 21 due to unrelated causes with no evidence of locoregional glottic disease and one due to laryngeal cancer. This was the patient who refused to undergo laryngectomy. The larynx was therefore preserved in all the 84 patients (100%).

**Fig. 2:**
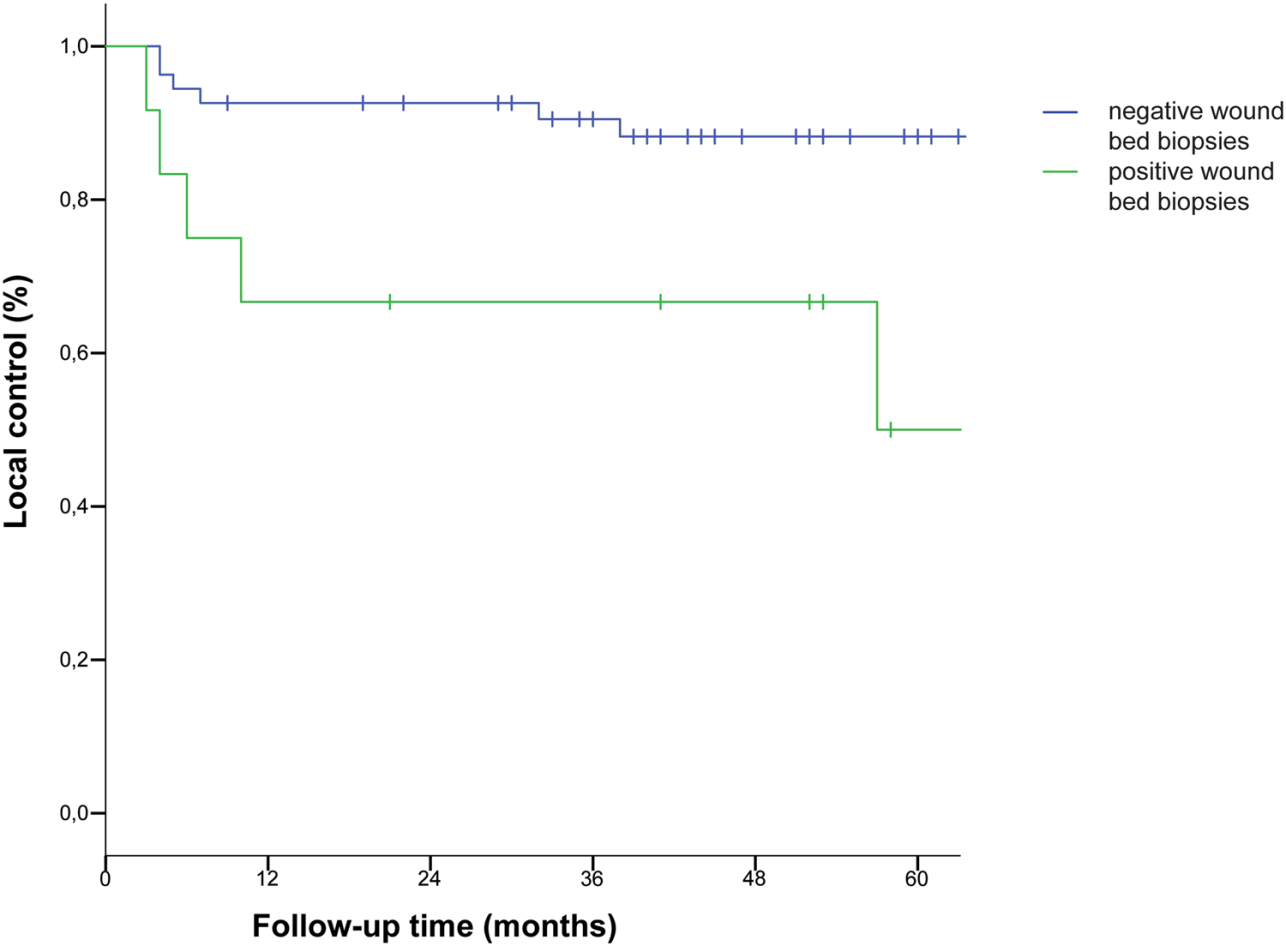
Kaplan–Meier curve for local control in relation to wound bed biopsies

The 5-year LC, OS, DSS and LP are 78.6, 78.0, 98.6, and 100%, respectively. In univariate analysis, positive wound bed biopsy was the only factor with significant impact on local control with a lower rate of local control for patients with wound bed biopsies positive for SCC (50 vs 88.2%, *p* = 0.009) (Table 2; Fig. 2). Resection margin status had no impact on local control (76.9 vs 85.9%, *p* = 0.469) (Table 2). The three other variables (pT-category, tumour differentiation, and number of biopsies) also had no impact on local control (Table 2).

**Table 2:** Univariate survival analysis for prognostic factors on local control

Variable	No. of patients	5-year local control (%)	*p* value*
Pathologic T category			0.543
Tis	19	78.9	
T1	50	80.4	
T2	15	70.0	
Resection margin			0.489
Not free	68	76.9	
Free	16	85.9	
Tumour differentiation			0.654
Poor	7	77.6	
Moderate	34	80.9	
Good	28	71.4	
Additional wound bed biopsies			0.009
Negative	54	88.2	
Positive	12	50.0	
Biopsy count			0.323
≤ 5	49	73.8	
≥ 6	35	84.5	

*Calculated with log-rank test

## Discussion

The objective of this study was to assess the impact of surgical margins and wound bed biopsies on local control in patients with early glottic cancer (Tis-T2) after treatment with TLM. In this study, margin status after TLM did not significantly influence local control. However, we found that when additional biopsies of the wound bed were positive for SCC, local control was significantly lower in these patients.

Studies on the role of surgical margins in predicting local control after TLM in early glottic carcinoma vary greatly in their recommendations. While some authors recommend re-treatment once positive margins are found [14, 26], other authors agree on the statement that instead of solely assessing margin status, the surgeon’s clinical judgment should be incorporated [10, 22, 27]. Perioperative evaluation of the radicality of the procedure by an experienced surgeon is considered an essential factor in decision-making by them. Sigston et al. [11] advocated a ‘wait and see’ policy, to avoid a large number of unnecessary procedures, pointing out that because of the readily accessible location of glottic cancers, qualitative visual follow-up is adequate for monitoring patients with early lesions. In moderately advanced lesions, however, periodical imaging should be added for early detection of submucosal recurrence [28]. Moreover, as the glottic area reveals symptoms earlier than other areas, enabling earlier detection by patients themselves they will visit their physicians outside the protocol if they experience symptoms. Later authors have also adopted this point of view [10, 22, 29]. In line with these aforementioned arguments, if following a wait-and-see policy, it is necessary to have compliant patients who will be available for follow-up by rigid or flexible endoscopy with stroboscopy combined with additional imaging techniques, such as narrow-band imaging. However, the ELS recommends second-look microsurgery in case of positive margins at histopathological assessment [21]. In our experience, however, there are some issues with routine second-look procedures. Firstly, during the healing stage of the vocal fold which takes up to 3 months evaluation is often compounded by wound debris, granuloma formation, and vulnerable mucosa. Once the mucosa is healed, in our experience flexible laryngoscopy with modern chip-on-tip cameras and now additional tools such as NBI closely matches the information gained during direct endoscopy. Palpation is missed, but in our experience, this is not entirely reliable and it is doubtful if a small submucosal residue will be detected at such an early stage. Also, in moderately advanced carcinomas where submucosal recurrence is more frequent imaging should be the primary modality for early detection or recurrences. Secondly, routine second-look procedures for positive surgical margin would lead to a significant number of unnecessary procedures under general anaesthesia, particularly in a population that is often affected by comorbidities. Therefore, based on our results and aforementioned arguments, we prefer a wait-and-see policy in case of positive surgical margins, with second-look procedures only on a case-by-case basis, depending on the surgeon’s evaluation of the procedure and possible healing abnormalities of the vocal fold and to add periodical imaging for early detection of submucosal recurrence in extended resections (type III or more) or on indication.

Since the surgical margins are carbonized after TLM, it is difficult to distinguish between positive and suspicious margins and in contrast to other studies we grouped suspicious and positive margins together and reported them as not free. This approach has also been reported by several other authors [30, 31]. The handling of surgical margins should be taken into consideration when comparing results with literature because the definition of positive and negative surgical margins varies between studies. Remarkably, 81.0% of the surgical margins were positive (or suspicious) in this study, while in the literature the rate of suspicious or positive margins on final examination ranged from 6 to 50% [12]. There may be several explanations for this. Although all specimens were reviewed for this study, they were scored in a binary way (positive or suspicions versus clearly negative). This binary scoring could be one of the reasons that we present higher percentages of (possibly) positive margins. Another reason could be that in the earlier years of the study some tumours were operated with a wider spot diameter (0.7 mm). In addition to the well-known problems of shrinkage and carbonization this could have further eroded the margin without compromising the radicality of the resection. Furthermore, we cannot exclude a local variation in evaluation protocol having led to this result, and finally we cannot exclude that the pathologist reviewing the samples may have been the particularly meticulous knowing that the results would be analysed for a study. Despite the high rate of positive margins, our data are in line with the literature, showing that positive margins have no impact on local control [8, 10, 11, 16].

To our knowledge, additional wound bed biopsies are not commonly performed. Some authors rightfully claim that sampling error can occur leading to false-negative results [16, 32]. However, seeing the results of this study, it is our opinion that although not a guarantee of a radical resection wound bed biopsies can help distinguish those patients at risk for recurrent disease whilst significantly lowering the number of unnecessary procedures that would result from routine re-resections of positive surgical margins.

To the best of our knowledge, there are only a few studies that have performed peroperative additional biopsies [17, 19]. In contrast with our study, Charbonnier et al. carried out additional biopsies when margins were clinically suspicious during the procedure. However, these biopsies were not routine and they also grouped positive additional biopsies with positive margins. Therefore, the prognostic value of their additional biopsies was not independently analysed [19]. Manola et al. performed wound bed biopsies but again, only when the margins were macroscopically positive or uncertain. In our study, additional biopsies were the best predictor of local control. However, not in all patients, additional biopsies were performed.

The 5-year LC for Tis, T1, and T2 in our study was 78.9, 80.4, and 70.0%, respectively. These rates are comparable with large series in literature, although the 5-year LC for T1 tumours is in the lower range [33]. A detailed analysis of our local control rates showed that the higher percentage of recurrence is related to a learning curve. The proportion of recurrences for the two senior laryngologists performing 92.9% of the procedures was 15.4%. The remaining 7.1% of procedures were performed by various less experienced surgeons and had a recurrence rate of 66.6% which had some impact on our overall 5-year rate of local control. This study, therefore, confirms earlier reports of the importance of the learning curve in TLM [34, 35]. In addition, not all patients with positive wound bed biopsies underwent a re-excision. It also seems that although no evidence of persistent carcinoma was found, the re-excisions were not always extensive enough. With this knowledge, we now perform more extensive re-resections in patients with positive wound bed biopsies. Our 5-year overall survival of 78.0% was slightly lower than in some other series in literature [36, 37]. This combined with the fact that four patients were lost to follow-up may also have had some small impact on our rate of local control as there were fewer life years included in the analysis.

Most recurrences (75%) were found within the first year, pointing out the importance of extensive follow-up and extra level of awareness in the first year after surgery. The majority of the recurrences in our study were treated with TLM, RT or TLM and RT. No laryngectomies were performed in case of recurrent disease. The only patient in whom this was indicated refused to undergo further treatment. This shows that if follow-up is carried out efficiently and recurrent tumours are treated in time, laryngectomy rate is low. The 5-year DSS and LP were in accordance with literature [24].

Due to the retrospective study design, this study has some limitations. First, although it was standard protocol, not all patients had additional wound bed biopsies taken during surgery due to several reasons. In some cases, surgeons were relatively certain of free surgical margins based on clinical evaluation and in some cases, the tumour was suspect for laryngeal papilloma. In three cases no cause was mentioned.

In summary, this study shows that additional wound bed biopsies can help predict recurrence after TLM for early glottic carcinoma in which ELS type I–III resection are performed and can help identify those patients where additional treatment is indicated. Looking at literature and at our own results, there is evidence to support a ‘wait and see’ policy in patients with positive surgical margins if the perioperative findings of an experienced surgeon as well as close follow-up are incorporated, although this conclusion has to be confirmed in additional studies in other, larger patients populations. This is further supported by the finding that treatment for recurrent disease in our population was highly successful and laryngectomy was indicated in only one patient (1.2%). For patients with positive wound bed biopsies, we strongly recommend considering additional treatment in the form of re-excision with TLM or RT as the chance of a recurrence is high (50%).
